# Defunctioning Ileostomy to Prevent the Anastomotic Leakage in Colorectal Surgery. The State of the Art of the Different Available Types

**DOI:** 10.3389/fsurg.2022.866191

**Published:** 2022-04-13

**Authors:** Diego Coletta, Cristina De Padua, Immacolata Iannone, Antonella Puzzovio, Paola Antonella Greco, Alberto Patriti, Filippo La Torre

**Affiliations:** ^1^Department of Surgical Sciences, Policlinico Umberto I University Hospital, Sapienza University of Rome, Rome, Italy; ^2^Department of General Surgery, Ospedali Riuniti Marche Nord, Pesaro, Italy; ^3^Department of General Surgery, Emergency Department, Emergency and Trauma Surgery Unit, Policlinico Umberto I University Hospital, Sapienza University of Rome, Rome, Italy

**Keywords:** defunctioning ileostomy, tube ileostomy, cannula ileostomy, loop ileostomy, colorectal surgery, anastomotic leak, fecal diversion

## Introduction

Defunctioning ileostomy (DI) is a surgical procedure adopted for fecal diversion in colorectal surgery to prevent the most important complication, i.e., anastomotic leakage (AL). It could be defined as a defect in the intestinal wall integrity at the suture site leading to a communication between the inside and outside compartments such as pelvic abscess close to the anastomosis and recto-vaginal fistula (1). Most surgeons suggest the use of fecal diversion in patients undergoing low anterior resections of rectal tumors followed by ultra-low colorectal or coloanal anastomoses at high risk for anastomotic failure. Although a stoma does not always prevent AL, it may reduce the incidence of sepsis in the event of leakage and decreases the rate of emergency reoperation (2–4). Fecal diversions have been associated with poor quality of life, stoma-related complications from 3 to 33%, and perioperative risk of stoma closure later on or a reversal of stoma not happening because of patients at high risk of complications (5). In the past decade, most techniques have been described as variants of the conventional loop ileostomy or as novel technical notes, changing the site of stoma or using tubes to perform it. With the advent of minimally invasive surgery, new techniques have been developed in an attempt to maintain the concept of less invasiveness for the patients. The aim of our paper is to give a snapshot of the current literature on the available types of DI to prevent AL in colorectal surgery, searching by three different electronic databases, namely Pubmed/Medline, Web of Science (WOS), and EMBASE, using a combination of the following MESH terms: “loop ileostomy,” “cannula ileostomy,” “tube ileostomy,” “defunctioning ileostomy,” “diverting loop ileostomy,” “colorectal surgery,” “anastomotic leak,” and “fecal diversion.” The references of the retrieved articles were screened to find further studies. We chose to not describe “Ghost ileostomy” and “Hidden ileostomy,” because these are not ostomies but considered as alternative procedures to DI, so cannot be included in the group of fecal diversionsa.

## “Turnbull” Loop Ileostomy

**Figure 1:**
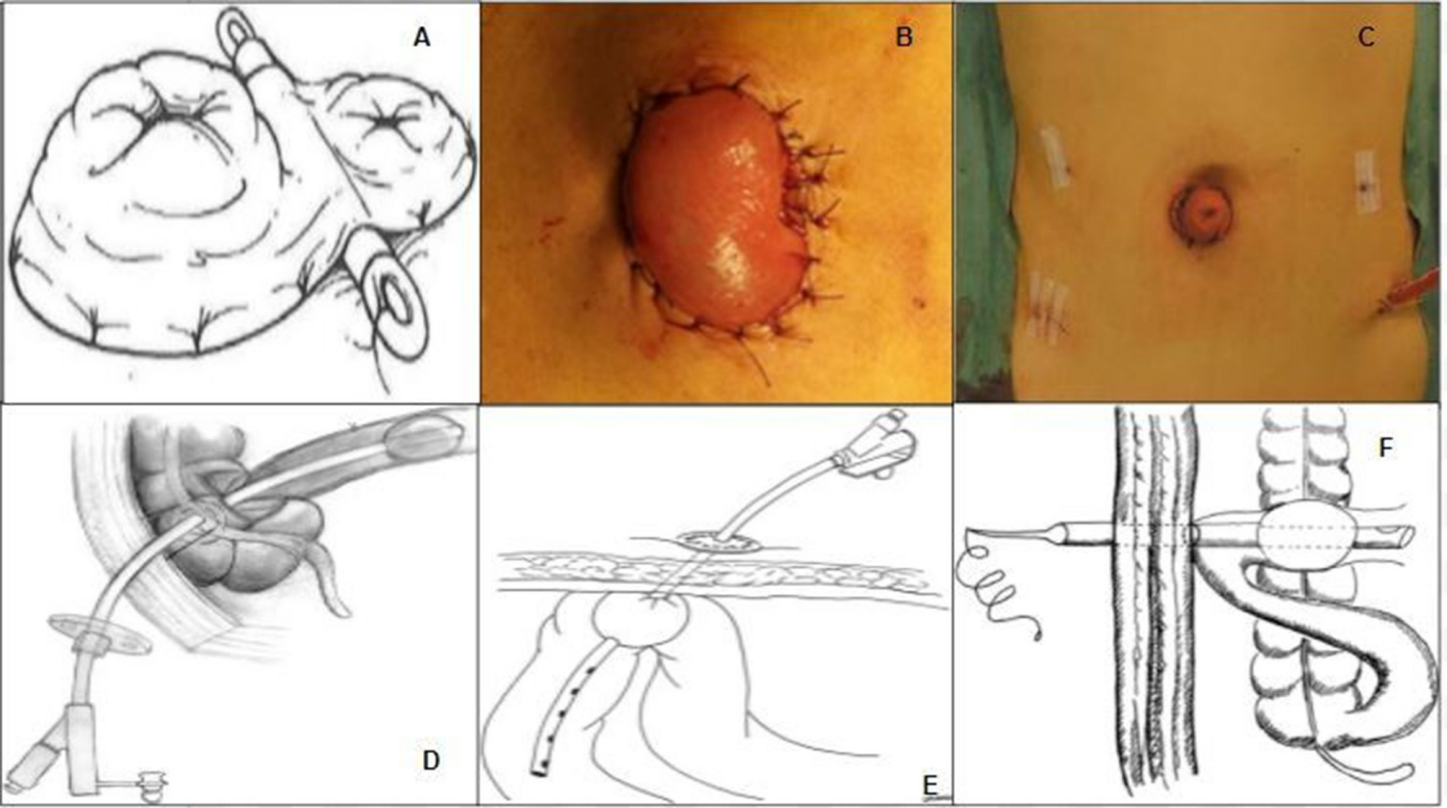
**(A)** Classic Turnbull loop ileostomy, from Whitehead and Cataldo (11). **(B)** Skin bridge loop ileostomy, from Ye et al. (12). **(C)** Umbilical ileostomy, from Eto et al. (13). **(D)** Transcaecal ileostomy, from Monzòn-Abad et al. (14). **(E)** Percutaneous ileostomy, from Rondelli et al. (15). **(F)** Cannula ileostomy from Hua et al. (16).

The more popular technique used to perform a conventional loop ileostomy is that described for the first time by Turnbull and Weakley (6) around the late 1960s. The intestinal loop is pulled out through an abdominal transparietal circular opening at the level of the right iliac fossa and fixed with four interrupted sutures between the parietal fascia, peritoneum, and seromuscular layer of the bowel, in order to avoid postoperative prolapse. A rod is used to pull out and keep the loop in place to avoid retraction and to exclude the efferent loop from the transit of the bowel content. After the opening of the stoma, interrupted mucocutaneous stitches with absorbable materials are used to complete stoma fixation to the skin. The ileostomy takedown is made with a peristomal skin incision, complete mobilization of the bowel loop, intestinal anastomosis, and abdominal wall reconstruction. In the past decades, many variants of classic techniques have been reported. The changes have consisted of eliminating the use of the rod, trying new material for the rod, suturing the efferent loop, and changing the site of ostomy (7–10) (Figure 1A).

## Skin Bridge Loop Ileostomy

This is the most recent variant of the classic loop ileostomy technique, consisting of using a skin flap as a rod (17). The skin is incised, creating a rectangular skin bridge about 3 cm long and 1 cm wide, and the subcutaneous fat is divided. This flap is passed through an avascular window opened in the mesentery at the apex of the chosen ileal loop and then secured with separate stitches of 2/0 absorbable suture to the distal edge of the opening, determining the exclusion of the efferent loop of the stoma. The afferent and the efferent loops are fixed to the skin with a 3/0 absorbable suture to prevent retraction and dislocation. Some recent papers (12, 18) demonstrate that the skin bridge loop ileostomy may significantly reduce the early postoperative stoma-related complications, the frequency of exchanged ostomy bags, and patient medical costs after hospital discharge if compared with a conventional loop ileostomy. The ostomy closure follows the same techniques of conventional loop ileostomy (Figure 1B).

## Umbilical Ileostomy

Used for the first time in pediatric patients with Hirschsprung's disease or imperforate anus (10), this technique has increased in use together with the rise of laparoscopic surgery in order to maintain the concept of minimal invasiveness of the surgical procedures. The loop of the ileum designated for the ileostomy was brought out without tension through the umbilical port site with a vertical skin incision just below the umbilicus. It is important to widen the fascial incision to allow for a 5 cm gap as for the conventional ileostomy, and the intestinal serosa and fascia were fixed. Three points of the serosal muscular layer were sutured on the caudal side and on both lateral sides to prevent retraction. The intestinal tract was opened, and the umbilicus is fixed to the incision end of the stoma to assist in the elevation of the intestinal tract. Ostomy reversal is performed through a full mobilization of the stoma including the umbilicus, followed by the anastomosis. Finally, the skin is fixed to the muscle layer subcutaneously with two needles to form a new umbilicus (19–25) (Figure 1C).

## Transcecal Ileostomy

Simpson and Srivastava (26) described this technique of ileal diversion in 1975 to allow a complete colonic lavage and ileal decompression in elective colonic surgery. A Foley catheter (26, 27) or a gastrostomy tube (14) is inserted through the cecum in the ileocecal valve and then the balloon is inflated. The catheter and the cecum are fastened with a single or double purse-string suture to the parietal peritoneum and abdominal wall. Ostomy closure is performed by a gradual deflation of the balloon started at postoperative day 5 (27) or at postoperative day 7 (14), and when is complete, the catheter is removed (Figure 1D).

## Percutaneous Ileostomy

After the performance of colorectal anastomosis, a modified 18 or 20 Fr jejunostomy tube is placed into the distal ileum about 40 cm proximal to the ileocecal valve by ensuring that the distal part of the tube was in the afferent loop to optimize the drainage (15). The jejunostomy balloon was inflated with 7–10 ml of normal saline then the catheter is fixed in the ileal loop with a purse-string and was brought out through the abdominal wall in the right inferior quadrant also by using a port incision in laparoscopic and robotic surgery. Between the 8th and the 11th postoperative days, a CT scan with a trans-anal enema of hydrosoluble iodate contrast is performed to assess the integrity of the anastomosis, and the catheter can be removed by deflating progressively the balloon. Finally, the abdominal orifice is kept open and connected to a urostomy bag (Figure 1E).

## Tube/Cannula Ileostomy

The endotracheal tube can be used to perform fecal diversion after the colorectal anastomosis; some authors named this technique “cannula” ileostomy (16) and some others “tube” ileostomy (28, 29). A double row of concentric purse-string sutures is placed onto the ileum wall with absorbable sutures and the tracheal cannula is inserted into the distal ileum through a small incision within the inner purse-string, after which the inner and then the external purse-string sutures are tied. Thereafter, normal saline is injected into the balloon and the tube is pulled out through the abdominal wall. The loop is secured to the same location at the parietal peritoneum, near the tube end with seromuscular stitches. The cannula is then pulled tight, and sutures at the fixation site are tightly knotted. The procedure ends with or without a reversible single row of staples across the whole width of the terminal ileum about 10 cm distal to the site of tube insertion. The tube is removed 2 days after the anal function of the patient resumes, during which the tube is blocked with the deflated balloon to ensure that the passage of bowel content continues after its removal between the 20th and the 75th postoperative days.

Chowdri et al. (30) described the same procedure but using a 26 Fr three-way self-retaining Foley catheter, and they also named it “tube ileostomy.” In the postoperative period, the management of ostomy requires a regular check of the free flow of contents by washing the tube with normal saline. The tube is deflated between the 5th and 7th postoperative days, clamped after the second week, and finally removed after the third week of surgery to obtain a controlled fistula (Figure 1F).

Table 1 shows the characteristics of the main included studies.

**Table 1:** Characteristics of the main included studies.

**References**	**Type of ileostomy or comparison**
Turnbull and Weakley (6)	Loop ileostomy
Flati et al. (7)	Loop ileostomy
Pace et al. (17)	Skin bridge loop ileostomy
Fitzgerald et al. (10)	Umbilical ileostomy
Hada et al. (19)	Umbilical ileostomy
Ishiguro et al. (20)	Umbilical ileostomy
Mushaya et al. (21)	Umbilical ileostomy
D'Alessaandro et al. (22)	Umbilical ileostomy
Miyo et al. (24)	Umbilical ileostomy
Seow-En et al. (25)	Umbilical ileostomy
Simpson and Srivastava (26)	Transcaecal ileostomy
Winslet et al. (27)	Transcaecal ileostomy
Monzòn-Abad et al. (14)	Transcaecal ileostomy
Rondelli et al. (15)	Percutaneous ileostomy
Hua et al. (16)	Cannula ileostomy
Sheng et al. (29)	Tube ileostomy
Chowdri et al. (30)	Tube ileostomy
Dzki et al. (8)	Loop ileostomy vs. skin bridge ileostomy
Carranante et al. (18)	Loop ileostomy vs. skin bridge ileostomy
Ye et al. (12)	Loop ileostomy vs. skin bridge ileostomy
Eto et al. (23)	Loop ileostomy vs. umbilical ileostomy
Eto et al. (31)	Loop ileostomy vs. umbilical ileostomy
Zhou et al. (28)	Loop ileostomy vs. tube ileostomy
Rondelli et al. (32)	Loop ileostomy vs. percutaneous ileostomy
Hanju et al. (33)	Loop ileostomy vs. cannula ileostomy

## Discussion

Defunctioning ileostomy is a surgical procedure adopted for the fecal diversion in colorectal surgery to prevent AL. The perfect technique to perform an ileostomy does not exist and any one of the available procedures could be best suited for the patient. All the techniques described could be adopted in open or minimally invasive surgery by using the laparoscopic port incisions adapted as needed, except for the umbilical ileostomy that can be performed only in laparoscopic surgery for obvious intrinsic technical reasons. We aimed to give a snapshot of the current literature on the available types of DI in colorectal surgery. The most important characteristic of a fecal diversion is to be really “defunctioning” as much as possible, without stoma-related complications and with only some or no discomfort for the patients. Moreover, a temporary ileostomy should be easy to take down spontaneously if possible, as described for some techniques (15, 16). In our study, we described the different available techniques that could not have been compared, but some have been compared to the conventional Turnbull loop ileostomy (8, 12, 18) in terms of stoma-related complications and ostomy management. Carannante et al. (18) compared the conventional technique with a plastic rod to the skin bridge one, showing an improvement of stomal infection, dermatitis, and ulcers in the second group. Besides, the average number of exchanged stoma wafers per week resulted in more than half with statistical significance. No studies investigated eventual differences in the ostomy take-down outcomes that seem to be the same for both techniques. Eto et al. (23, 31) compared the conventional ileostomy and umbilical ileostomy after the laparoscopic anterior resection for rectal cancer. The studies demonstrated a lower wound infection rate in the group of conventional loop ileostomy with better surgical outcomes, but a significantly lower incidence of incisional hernia and relative risk for its development in the umbilical ileostomy group. The temporary percutaneous ileostomy seems to be a valid alternative to the classic loop ileostomy after low anterior resection and extraperitoneal anastomosis, offering a more comfortable and complete fecal diversion with fewer stoma-related and surgical complications if compared with a conventional DI (32). The real novelty is that this ostomy does not require surgery for its closure. The comparison between tube/cannula ileostomy and conventional loop ileostomy has shown no statistical difference in terms of anastomotic dehiscence, stomal complications, and pain. The main differences are the longer hospital stay for the traditional loop ileostomy group and the need for a second surgery for its closure (28, 33). In daily practice, no one technique leads to superior performance than another, and no evidence supports to advise the use of one routinely; the confidence and the expertise of the surgeons in performing a DI and the characteristics of patients play a key role in the choice of the technique to adopt. Further prospective studies with multiple arms of investigation are needed to compare the different techniques of DI to prevent AL in colorectal surgery.

## Conclusion

The perfect technique to perform a DI does not exist; different techniques can be performed and every patient should receive the proper tailored one. The surgeon should know every one of these available choices and use them as the arrows in the quiver of an archer when needed.

## Author Contributions

DC and FLT have designed the study. DC, II, and CD have performed the literature search and extracted data. DC has written the manuscript and is responsible for the financial support. FLT, APu, APa, and PAG have critically revised the manuscript. All authors contributed to the article and approved the submitted version.

## Funding

DC received financial aid for the drawing up of the present article from the Ph.D. Course in Advanced Surgical Technologies of the Sapienza University of Rome.

## Conflict of Interest

The authors declare that the research was conducted in the absence of any commercial or financial relationships that could be construed as a potential conflict of interest.

## Publisher's Note

All claims expressed in this article are solely those of the authors and do not necessarily represent those of their affiliated organizations, or those of the publisher, the editors and the reviewers. Any product that may be evaluated in this article, or claim that may be made by its manufacturer, is not guaranteed or endorsed by the publisher.
